# Causal relationship between particulate matter and COVID-19 risk: A mendelian randomization study

**DOI:** 10.1016/j.heliyon.2024.e27083

**Published:** 2024-02-24

**Authors:** Jiayi Zhu, Yong Zhou, Qiuzhen Lin, Keke Wu, Yingxu Ma, Chan Liu, Na Liu, Tao Tu, Qiming Liu

**Affiliations:** a Department of Cardiovascular Medicine, The Second Xiangya Hospital, Central South University, Changsha, Hunan 410011, PR China; b International Medical Department, The Second Xiangya Hospital, Central South University, Changsha, Hunan 410011, PR China

**Keywords:** COVID-19, PM2.5, Air pollution, Particulate matter, Mendelian randomization

## Abstract

**Background:**

Observational studies have linked exposure to fine (PM2.5) and coarse (PM10) particulate matter air pollution with adverse COVID-19 outcomes, including higher incidence and mortality. However, some studies questioned the effect of air pollution on COVID-19 susceptibility, raising questions about the causal nature of these associations. To address this, a less biased method like Mendelian randomization (MR) is utilized, which employs genetic variants as instrumental variables to infer causal relationships in observational data.

**Method:**

We performed two-sample MR analysis using public genome-wide association studies data. Instrumental variables correlated with PM2.5 concentration, PM2.5 absorbance, PM2.5-10 concentration and PM10 concentration were identified. The inverse variance weighted (IVW), robust adjusted profile score (RAPS) and generalized summary data-based Mendelian randomization (GSMR) methods were used for analysis.

**Results:**

IVW MR analysis showed PM2.5 concentration [odd ratio (OR) = 3.29, 95% confidence interval (CI) 1.48–7.35, *P*-value = 0.0036], PM2.5 absorbance (OR = 5.62, 95%CI 1.98–15.94, *P*-value = 0.0012), and PM10 concentration (OR = 3.74, 95%CI 1.52–9.20, *P*-value = 0.0041) increased the risk of COVID-19 severity after Bonferroni correction. Further validation confirmed PM2.5 absorbance was associated with heightened COVID-19 severity (OR = 6.05, 95%CI 1.99–18.38, *P*-value = 0.0015 for RAPS method; OR = 4.91, 95%CI 1.65–14.59, *P*-value = 0.0042 for GSMR method) and hospitalization (OR = 3.15, 95%CI 1.54–6.47, *P*-value = 0.0018 for RAPS method). No causal links were observed between particulate matter exposure and COVID-19 susceptibility.

**Conclusions:**

Our study established a causal relationship between smaller particle pollution, specifically PM2.5, and increased risk of COVID-19 severity and hospitalization. These findings highlight the importance of improving air quality to mitigate respiratory disease progression.

## 1 Introduction

The global Coronavirus disease 2019 (COVID-19) pandemic, caused by the highly contagious severe acute respiratory syndrome coronavirus 2 (SARS-CoV-2), is exacting a catastrophic impact on humanity. According to the latest World Health Organization (WHO) report as of April 17, 2023, there have been 762,791,152 confirmed cases of COVID-19 worldwide, resulting in 6,897,025 deaths [1]. In response, medical and public health professionals are actively identifying potential risk factors to better control the transmission and adverse consequences of COVID-19 in high-risk populations. Observational studies have identified several clinical conditions that may heighten COVID-19 susceptibility and severity, including cardiovascular diseases, chronic pulmonary disease, and other comorbidities [2,3]. Recent research has established a connection between exposure to air pollution, specifically PM2.5, and a rise in COVID-19-related mortality [4,5], highlighting the potential impact of air pollution on disease progression.

Airborne particulate matter (PM), which is widely regarded as one of the most common and significant constituents of air pollution, has been associated with increased mortality from various diseases [6]. These particles are classified based on their aerodynamic diameters. PM2.5, also known as fine particulate matter, is a complex mixture comprising a variety of organic and inorganic substances. The primary components of PM2.5 mass include organic carbon, elemental carbon, nitrate, sulfate, ammonium, and organic markers such as polycyclic aromatic hydrocarbons [7]. PM2.5 has a significant impact as its small size enables it to penetrate deeply into the lung. This particular characteristic provides a foundation for modifying the lung's vulnerability to inflammation and eliciting intense immune responses upon infection [8]. Previous studies have proposed that long-term exposure to higher levels of PM2.5 and PM10 could increase the incidence and mortality rate of acute respiratory distress syndrome (ARDS) and COVID-19 [9,10]. In Rome, Italy, a 16-month study conducted with a total of 1,594,308 participants revealed no correlation between PM2.5 concentration and COVID-19 incidence [hazard ratio (HR) = 1.01, 95% confidence interval (CI) 0.99–1.03], but a significant correlation with COVID-19-related death (HR = 1.08, 95%CI 1.03–1.13) [5]. Similarly, Gupta et al. analyzed air pollution and COVID-19 cases in 9 Asian cities, and the result showed that PM2.5 concentration is related with COVID-19 death (*P* = 0.033), but not PM10 concentrations (*P* = 0.118) [11]. The effect of air pollution on COVID-19 related outcomes remained controversial among different locations and populations, possibly due to insufficient sample size and the existence of confounding factors. The most contentious aspects involve the influence of particulate matter with varying diameters on COVID-19 progression and the impact of particulate matter on different outcomes of COVID-19. Therefore, the causal relationship between particulate matter exposure and COVID-19 incidence and severity still requires further investigation and clarification.

Randomized controlled trial (RCT) is typically the preferred method for exploring causal relationships. However, they may be unfeasible due to ethical and budgetary restrictions. Meanwhile, the Mendelian randomization (MR) study serves as an alternative tool to explore causal relationships, utilizing genetic variants, such as single nucleotide polymorphisms (SNPs), as instrumental variables (IVs) to estimate causal inference between exposures and outcomes. Since germline genetic variants are assorted randomly at conception, they can be considered independent of environment and self-adapted behaviours [12], thus minimizing the influence of confounders which are unavoidable even in RCT experiments. In this study, we conducted the first causal investigation by performing a two-sample MR analysis to determine the relationships between exposure to various airborne particulate matter traits (PM2.5 concentration, PM2.5 absorbance, PM2.5-10 concentration, and PM10 concentration) and COVID-19 outcomes (susceptibility, hospitalization, and severity) utilizing publicly accessible genome-wide association studies (GWAS) summary data. This study assessed the impact of particulate matter exposure on the risks associated with COVID-19 and laid the groundwork for appreciating the benefits of improved air quality on human health concerns.

## 2 Methods

### 2.1 Study population

The summary-level airborne particulate matter exposure data were derived from the ESCAPE project (European Study of Cohorts for Air Pollution Effects), which included 423,796 European participants. The project estimated average pollution particle concentrations between late 2008 and early 2010 using a land use regression algorithm [13]. PM2.5 and PM10 refer to airborne particles with diameters of 2.5 μm or less and 10 μm or less, respectively. Their concentrations were calculated by dividing the mass of collected particles by the sample volume. PM2.5-10 concentrations were derived by subtracting PM2.5 concentration from PM10. PM2.5 absorbance was calculated based on relative reflectance of the sample filter measured with smoke stain reflectometer, the stained filter's area, and the sampled volume [14]. For more detailed measurement protocols and calculation formulas, please consult the ESCAPE project website (http://www.escapeproject.eu/manuals/). Datasets were downloaded from the publicly available dataset MRC IEU OpenGWAS website (https://gwas.mrcieu.ac.uk/datasets). The exposure dataset IDs were ukb-b-10817 for PM2.5 concentration, ukb-b-11312 for PM2.5 absorbance, ukb-b-12963 for PM2.5-10 concentration, and ukb-b-18469 for PM10 concentration.

COVID-19 summary datasets were obtained from COVID-19 Host Genetics Initiative [15], and the most recent and extensive release version round 7 (April 8, 2022) was selected for further analysis (https://covid19hg.org/results/r7/). We chose genetic association data from a total of 2,597,856 European participants. Three clinical indicators, namely 'Covid vs. population', 'hospitalized covid vs. population', and 'very severe respiratory confirmed covid vs. population', were chosen to represent COVID-19 susceptibility, hospitalization, and severity, respectively. The sample size and case proportion for each outcome are provided in detail in Supplementary Table 1.

### 2.2 Instrumental variables selection

In our study, we employed SNPs as IVs. A SNP represents a genetic variation where a single nucleotide base is replaced by another nucleotide. The utilization of SNPs as IVs is grounded in Mendel's law of independent assortment, which states that SNPs are randomly assorted at conception. This randomness makes them well-suited for serving as instrumental variables in our analysis. Eligible IVs were selected following three key assumptions: (1) SNPs should be significantly associated with exposure; (2) SNPs should not be linked to any potential confounder that could influence the relationship between exposure and outcome; (3) SNPs should affect the outcome solely through their influence on the exposure. A primary *P*-value threshold of 5 × 10^−8^ was utilized to ensure that SNPs exhibited genome-wide level significance with the exposures. For those without any SNPs reaching this level of significance, a more suggestive significant level (1 × 10^−6^) was applied. Furthermore, we used the European 1000 Genomes reference panel to eliminate any SNP with linkage disequilibrium (LD) using PLINK program (clumping cut-off distance window 10,000 kb, LD coefficient r^2^ < 0.001, version 1.9). The F-statistic represented the strength of the correlation between SNPs and exposures. An F-statistic greater than ten is considered less likely to cause weak instrument bias.

### 2.3 Mendelian randomization analysis

We conducted MR analysis to investigate the causal association between air pollution exposure and COVID-19 outcomes using the 'TwoSampleMR' package in R. MR analyses are analogous to RCTs when inferring causality [16]. The inverse variance weighted (IVW) meta-analysis method was chosen as the primary MR analysis, as it provides optimum accuracy with decent IV quality [17]. If sensitivity analyses indicated potential heterogeneity, a random effect model of the IVW method was employed; otherwise, a fixed effect method was used [18,19]. Different supplementary methods, namely MR-Egger, weighted median, simple mode, weighted mode, and maximum likelihood were utilized to verify the causal association. The intercept of the MR-Egger regression can represent horizontal pleiotropy, and it can be applied in the presence of unbalanced pleiotropy [20]. The weighted median method continues to offer a consistent estimate of the causal effect, even when over 50% of the instrumental variables are deemed invalid [21]. To further strengthen the reliability of the MR results, the robust adjusted profile score (RAPS) and the generalized summary data-based Mendelian randomization (GSMR) analysis were additionally employed. These methods provide robust estimates, accounting for potential pleiotropy and increasing confidence in our findings.

### 2.4 Sensitivity analyses

To further ensure IV quality, extensive sensitivity analyses were performed to ensure three key assumptions were met. We used Cochran's Q test to evaluate heterogeneity between each IV. MR-Egger regression was employed to assess pleiotropy, with an MR-Egger intercept indicating its existence. MR pleiotropy residual sum and outlier (MR-PRESSO) method is also a powerful complement for testing pleiotropy in MR, with the global test detecting horizontal pleiotropy, and the outlier test can correct the estimate by removing outliers if necessary [22]. The heterogeneity in dependent instrument (HEIDI) approach can also examine potential pleiotropy of IVs based on the GSMR analysis [23]. Furthermore, we utilized the PhenoScanner database (http://www.phenoscanner.medschl.cam.ac.uk/) to evaluate the potential associations between the selected IVs and any confounding factors that could potentially impact COVID-19 outcomes [24]. A leave-one-out analysis was conducted to identify potentially influential SNPs with significant effects on remaining IVW results.

### 2.5 Statistical analysis

All analyses were performed using R software (version 4.1.2). The 'MRPRESSO' package (version 1.0) was used for the MR-PRESSO method, 'mr.raps' package (version 0.2) for the RAPS method, and 'gsmr' package (version 1.1.0) for the GSMR method. An online web tool (https://sb452.shinyapps.io/power/) was utilized for power analysis. A *P*-value <0.05 were considered statistically significant in the primary analysis. A Bonferroni-corrected significance threshold of *P*-value <0.0042 (α' = 0.05/[3 × 4]) was used as a supplement to minimize false positive error further.

## 3 Results

### 3.1 Selection of IVs from particulate matter exposure datasets

We identified eight, five, seven, and eight independent SNPs for PM2.5 concentration, PM2.5 absorbance, PM2.5-10 concentration, and PM10 concentration, respectively. PM2.5–10 and PM10 summary data contain no SNPs meeting the primary significance criterion; hence a more suggestive significant level (1 × 10^−6^) was employed for these two exposure datasets. The characteristics of IVs screened for further MR analysis are listed in Supplementary Table 2. Given the current sample size and proportion of phenotype explained, our study has sufficient power to detect the observed effect on COVID-19 severity and hospitalization (Supplementary Table 1).

### 3.2 The causal effect of particulate matter exposure to COVID-19 outcomes

**Fig. 1:**
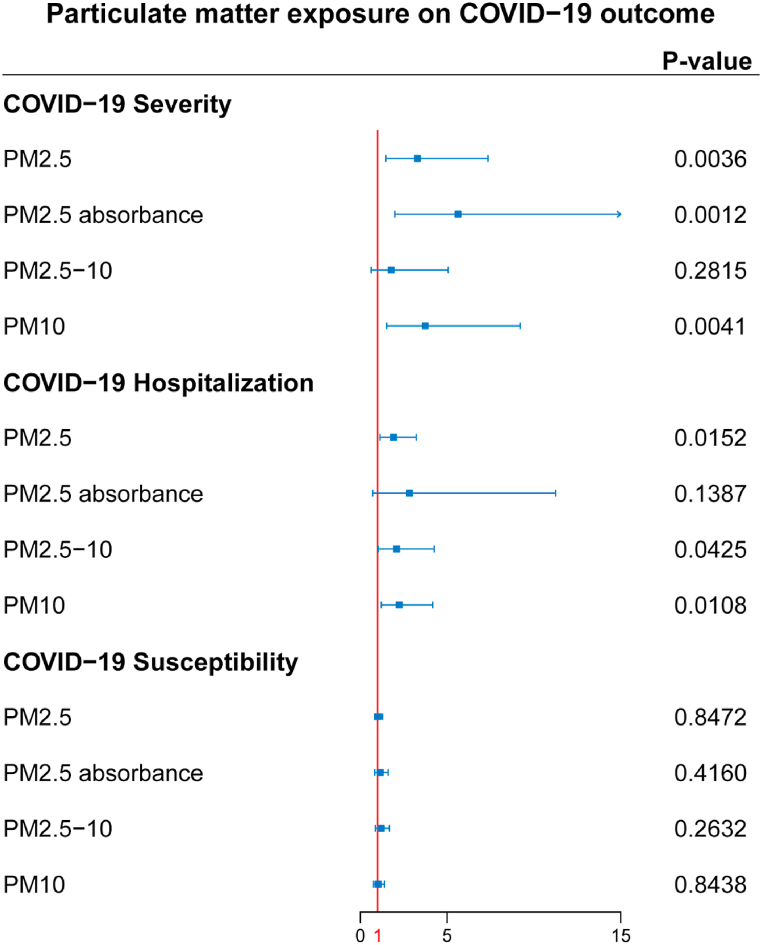
**The causal effect of each particulate matter exposure on COVID-19 severity, hospitalization, and susceptibility risks, assessed by the inverse variance weighted (IVW) method.***P*-value <0.0042 was considered significant for multiple testing mendelian randomization analysis using the IVW method.

**Fig. 2:**
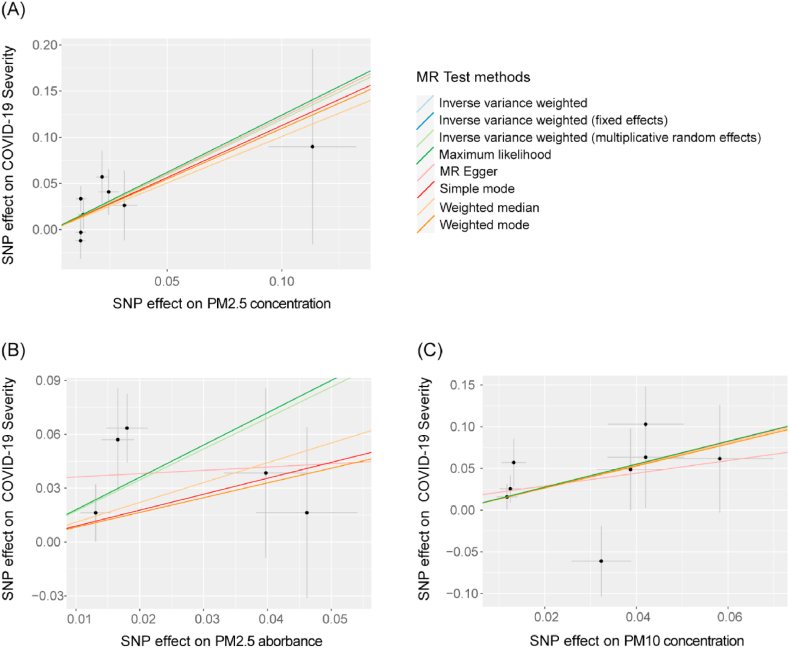
**Scatter plots of significant causality of the particulate matter exposure and COVID-19 outcomes.** Individual estimates about the causal impact of (A) PM2.5 concentration, (B) PM2.5 absorbance, and (C) PM10 exposure on COVID-19 severity. The x-axis shows the SNP effect on each exposure, and the y-axis shows the SNP effect on COVID-19 severity. Each line represents a different method used in mendelian randomization analysis. The positive slope of the lines indicated a positive correlation between exposures and outcomes. SNP, single-nucleotide polymorphism.

Complete MR results are shown in Supplementary Tables 3–6. Among four pollution exposures, IVW estimates that PM2.5 concentration (OR = 3.29, 95%CI 1.48–7.35, *P*-value = 0.0036), PM2.5 absorbance (OR = 5.62, 95%CI 1.98–15.94, *P*-value = 0.0012), and PM10 concentration (OR = 3.74, 95%CI 1.52–9.20, *P*-value = 0.0041) can significantly increase risk of COVID-19 severity. All three particulate matter exposures still had significant associations with COVID-19 severity after Bonferroni correction (*P*-value <0.0042). The concentration of PM2.5 (OR = 1.91, 95%CI 1.13–3.22, *P*-value = 0.015), PM2.5-10 (OR = 2.09, 95%CI 1.03–4.26, *P*-value = 0.042), and PM10 (OR = 2.24, 95%CI 1.21–4.17, *P*-value = 0.011) are associated with a significantly increasing risk of COVID-19 hospitalization; however, this significance disappeared after applying Bonferroni correction. No causal link was detected by IVW between particulate matter exposures and COVID-19 susceptibility (*P*-value = 0.85, 0.42, 0.26, and 0.84 for each exposure) (Fig. 1, Table 1). The ORs in all seven models (fixed effects IVW, multiplicative random effects IVW, MR-Egger, weighted median, simple mode, weighted mode, and maximum likelihood) demonstrated the same direction, as evidenced in the positive slopes of all the lines between genome-wide significant exposures and COVID-19 severity (Fig. 2A–C).

**Table 1:** **Causal effects assessed by IVW and sensitivity analysis for particulate matter exposure on COVID-19 outcomes.** IVW, inverse variance weighted. MR-PRESSO, mendelian randomization pleiotropy residual sum and outlier.

Exposure	Outcome	OR	95%CI	P-value	MR-Egger's P-value	Cochran Q test's P-value	MR-PRESSO P-value
PM2.5 concentration	Severity	3.29	1.48–7.35	0.0036^a^	0.972	0.464	0.527
	Hospitalization	1.91	1.13–3.22	0.015	0.835	0.256	0.285
	Susceptibility	1.02	0.82–1.28	0.85	0.634	0.470	0.485
PM2.5 absorbance	Severity	5.62	1.98–15.94	0.0012^a^	0.328	0.187	0.258
	Hospitalization	2.83	0.71–11.25	0.14	0.353	0.003^b^	0.023^b^
	Susceptibility	1.15	0.82–1.60	0.42	0.340	0.087	0.140
PM2.5-10 concentration	Severity	1.78	0.62–5.06	0.28	0.384	0.693	0.677
	Hospitalization	2.09	1.03–4.26	0.042	0.385	0.395	0.422
	Susceptibility	1.21	0.87–1.68	0.26	0.195	0.095	0.134
PM10 concentration	Severity	3.74	1.52–9.20	0.0041^a^	0.538	0.220	0.276
	Hospitalization	2.24	1.21–4.17	0.011	0.263	0.063	0.109
	Susceptibility	1.03	0.76–1.39	0.84	0.360	0.209	0.252

a *P*-value <0.0042 for mendelian randomization analysis using IVW method.

b *P*-value <0.05 for sensitivity analysis.

**Fig. 3:**
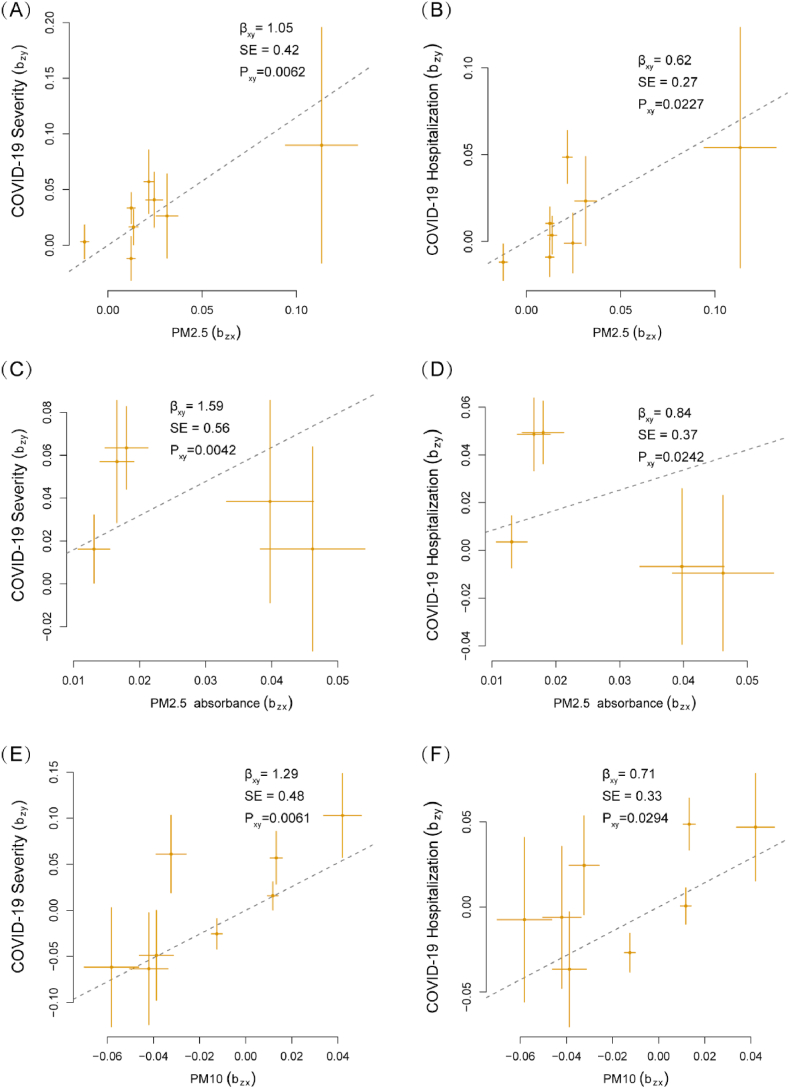
**Generalized summary Mendelian randomization (GSMR) analysis of particulate matter exposure and COVID-19 outcomes.** The plots report the linear relationship between the exposure estimates (b_zx_) and the outcome estimates (b_zy_). The b_xy_ represents the causal relationship between each exposure and outcome, SE is the standard error of the estimates, and P_xy_ is the P-value of the causal estimates.

As relaxed *P*-value thresholds could result in pleiotropy and weak instrument bias [25], the RAPS and GSMR methods are better suited to address these potential shortcomings. RAPS models revealed that PM2.5 concentration (OR = 3.37, 95%CI 1.45–7.86, *P*-value = 0.0048 for severity, and OR = 1.95, 95%CI 1.12–3.37, *P*-value = 0.017 for hospitalization), PM2.5 absorbance (OR = 6.05, 95%CI 1.99–18.38, *P*-value = 0.0015 for severity, and OR = 3.15, 95%CI 1.54–6.47, *P*-value = 0.0018 for hospitalization), and PM10 concentration (OR = 3.97, 95%CI 1.52–10.33, *P*-value = 0.0048 for severity, and OR = 2.35, 95%CI 1.22–4.52, *P*-value = 0.010 for hospitalization) is associated with increased risk of COVID-19 severity and hospitalization, and PM2.5-10 is associated with a weaker significance level concerning COVID-19 hospitalization (OR = 2.15, 95%CI 1.01–4.54, *P*-value = 0.046) (Table 2). The GSMR analysis verified that only PM2.5 concentration (OR = 3.16, 95%CI 1.39–7.20, *P*-value = 0.0062 for severity, and OR = 1.86, 95%CI 1.09–3.16, *P*-value = 0.023 for hospitalization), PM2.5 absorbance (OR = 4.91, 95%CI 1.65–14.59, *P*-value = 0.0042 for severity, and OR = 2.32, 95%CI 1.12–4.84, *P*-value = 0.024 for hospitalization), and PM10 concentration (OR = 3.63, 95%CI 1.42–9.26, *P*-value = 0.0069 for severity, OR = 2.04, 95%CI 1.07–3.88, *P*-value = 0.029 for hospitalization) had significant risk on COVID-19 severity and hospitalization (Fig. 3A–F, Table 2). After Bonferroni-correction, only PM2.5 absorbance is correlated with severity and hospitalization of COVID-19 in the RAPS method, whereas using the GSMR method, only the causal link between PM2.5 absorbance and COVID-19 severity can meet this level of significance criteria (*P*-value <0.0042). Both RAPS and GSMR methods further confirmed that exposure to air pollution does not lead to increased susceptibility to COVID-19.

**Table 2:** **Causal effects estimated by RAPS and GSMR method for particulate matter exposure on COVID-19 outcomes.** GSMR, generalized summary data-based Mendelian randomization. RAPS, robust adjusted profile score.

Exposure	Outcome	RAPS			GSMR		
		OR	95%CI	P-value	OR	95%CI	P-value
PM2.5 concentration	Severity	3.37	1.45–7.86	0.0048	3.16	1.39–7.20	0.0062
	Hospitalization	1.95	1.12–3.37	0.017	1.86	1.09–3.16	0.023
	Susceptibility	1.02	0.81–1.29	0.85	1.02	0.81–1.28	0.87
PM2.5 absorbance	Severity	6.05	1.99–18.38	0.0015^a^	4.91	1.65–14.59	0.0042^a^
	Hospitalization	3.15	1.54–6.47	0.0018^a^	2.32	1.12–4.84	0.024
	Susceptibility	1.15	0.82–1.63	0.41	1.14	0.81–1.60	0.46
PM2.5-10 concentration	Severity	1.80	0.60–5.42	0.29	1.74	0.60–5.04	0.31
	Hospitalization	2.15	1.01–4.54	0.046	1.94	0.93–4.04	0.075
	Susceptibility	1.22	0.87–1.72	0.25	1.17	0.84–1.65	0.35
PM10 concentration	Severity	3.97	1.52–10.33	0.0048	3.63	1.42–9.26	0.0069
	Hospitalization	2.35	1.22–4.52	0.010	2.04	1.07–3.88	0.029
	Susceptibility	1.03	0.76–1.41	0.84	1.03	0.76–1.39	0.87

a *P*-value <0.0042.

### 3.3 Sensitivity analysis

The Cochran's Q test detected no heterogeneity except for the causal estimate between PM2.5 absorbance and COVID-19 hospitalization (*P*-value = 0.003), therefore a random effect model IVW method was used in this case. The HEIDI-outlier method did not remove any SNPs, and no pleiotropy was detected using the MR-Egger intercept (Table 1). However, MR-PRESSO global tests indicated the existence of pleiotropy in the relationship between PM2.5 absorbance and COVID-19 hospitalization (*P*-value = 0.023) and identified an outlier IV. Despite this, removing the outlier did not change the direction and significance of the association assessed by the IVW method (OR = 1.69, 95%CI 0.44–6.52, *P*-value = 0.4497). The leave-one-out analysis also determined that no IV substantially influence overall MR analysis results for all three significant exposure-outcome estimates (Supplementary Fig. 1). All SNPs passed the correlation strength threshold, as the minimum F-statistic for four exposures were 30.04, 29.91, 24.44, and 24.42, indicating unlikely weak instrument bias for MR analysis (Supplementary Table 2). The MR results in our study were relatively robust, indicating that PM2.5 exposure is a risk factor for COVID-19 severity.

## 4 Discussion

In this study, we established causal estimates between several air pollution exposures and COVID-19 outcomes using large-scale GWAS datasets. Multiple statistical models, such as IVW, RAPS, and GSMR, have consistently suggested that PM2.5, particularly PM2.5 absorbance, can increase the risk of COVID-19 severity. This result remains valid after multiple test corrections of significance level. To the best of our knowledge, this is the first MR study that investigates the causal relationship between air pollution exposure and the outcomes of COVID-19. It could enhance our knowledge on how air pollution affects the severity of respiratory diseases including COVID-19.

PM2.5 can be inhaled into the lungs without being efficiently filtered by nasal hairs or mucus, implying a potential association between increased PM2.5 inhalation and the aggravation of the respiratory disease. Several epidemiological studies have illustrated that air pollution is a significant risk factor for COVID-19. A two-year observation study in the European area indicated the mortality of COVID-19 had positive correlation relationship with PM2.5 concentration, and a rise in COVID-19 mortality was found to follow each long peak of monitored PM2.5 concentration. An increase of 10.5 ± 2.5 % in COVID-19 mortality per 1 μg/m^3^ increase of PM2.5 concentration was observed in this study [9]. Similar findings were observed in a meta-analysis of 18 studies, which revealed that an increase in PM2.5 concentration led to a significant increase (OR = 2.27 per 10 μg/m³ change, 95% CI 1.41–3.66) in COVID-19 hospitalizations or critically ill patients [26]. The findings of our study are consistent with previous research indicating an elevated risk of hospitalization and/or mortality in COVID-19 patients linked to exposure to increased PM2.5 concentration. In terms of COVID-19 epidemic transmission, a one-year observation study suggested that the impact of PM2.5 on the COVID-19 infection rate varied monthly rather than remaining consistent throughout a year, partly due to its transmission being mainly dependent on human activity [27]. In Jiangsu province, China, total COVID-19 cases correlated positively with PM2.5, PM10, and NO_2_ levels, and negatively with O_3_ concentration. However, due to China's stringent lockdown policies, a direct causal relationship between pollution and epidemic prevalence is complex to infer [28]. Despite this, certain studies proposed that the impact of environmental temperature and air pollution on viral infectivity outweighs the influence of population density, thereby emphasizing the significant role of meteorological parameters over human transmission in the spread of the virus [[29], [30], [31][29], [30], [31][29], [30], [31]]. Addressing the controversy arising from these studies is challenging due to the inherent limitations of observational studies. Using MR methods, we found that although some study results indicated the COVID-19 infection rate is related to PM2.5 concentration, our MR analysis ruled out the causal association between particulate matter exposure and COVID-19 susceptibility. The effect of air pollution exposure to COVID-19 susceptibility could be negligible given the high infectivity of the SARS-CoV-2 virus, or influenced by the low detection rate in asymptomatic virus carriers [32].

Conventional epidemiological studies often yield unreliable causal estimates due to the interference of confounding factors. To overcome this limitation, we conducted an MR analysis in this study. Our MR analysis reinforced the possibility that an increase in particulate matter concentration can contribute to more severe manifestations of COVID-19 disease (as shown in the IVW method). However, using two more advanced and refined methods, we highlighted the influence of PM2.5 absorbance rather than particulate matter concentration. Previous studies have established a strong association between the absorbance of PM2.5 and traffic-related pollution emissions. Additionally, it showed a significant correlation with NO_2_ and NO_x_ levels (with R^2^ values of 0.80 and 0.85, respectively) but not the PM2.5 concentration [14]. Although no studies have specifically examined the impact of PM2.5 absorbance on COVID-19, several investigations have suggested that exposure to traffic-related air pollution was associated with elevated severity and mortality of COVID-19, possibly due to NO_2_ exposure [[33], [34], [35]]. Further research is required to verify the impact of PM2.5 absorbance on COVID-19 outcomes in large cohorts and to compare the predictive value between the PM2.5 concentration and absorbance.

While our research outcomes primarily focused on the physical health impacts of SARS-CoV-2 infection, it is also important to consider the psychological ramifications of the COVID-19 pandemic. A study examining anxiety rates among teachers in China during the COVID-19 epidemic found a prevalence rate of 13.67% [36]. This underlines the substantial mental health burden of the pandemic. Interestingly, long-term exposure to air pollution was also proposed to increase the risk of depression and anxiety [37]. However, it is yet to be scrutinized whether exposure to environmental factors has an effect on psychological outcomes and other aspects of COVID-19. Investigating the interplay between environmental factors and psychological resilience may help identify strategies for mitigating the health burden associated with the COVID-19 pandemic.

The mechanisms by which PM2.5 causes deteriorating COVID-19 conditions remain largely unknown. Particulate matter with aerodynamic diameters less than 2.5 μm can readily travel through the respiratory tract to reach type II alveolar cells, where SARS-CoV-2 can bind to angiotensin‐converting enzyme 2 (ACE2) receptor to infect the cells. A murine study indicated that PM2.5 disrupted the balance between ACE and ACE2 in the liver and brain, consequently diminishing the protective effects of the ACE2/Angiotensin 1-7/MAS pathway [38]. Additionally, PM2.5 exposure was observed to increase the expression of cyclooxygenase-2 (COX-2) in both lung and heart. This upregulation can trigger the activation of the NOD-like receptor family pyrin domain-containing 3 (NLRP3) inflammasome, potentially resulting in excessive inflammatory responses during a severe SARS-CoV-2 infection [39,40]. Additionally, PM2.5 can affect adaptive immune response. Studies have shown that PM2.5 exposure can induce an allergic immune reaction in a mice asthma model. This exposure disrupted the balance between allergic T-helper 2 cells (T_H_2) and T-helper 17 cells (T_H_17), and the anti-viral function of T-helper 1 cells (T_H_1). Consequently, the adaptive viral defence system is hampered, potentially leading to dysregulated immunopathological process in COVID-19 [41,42]. Exposure of PM2.5 has been associated with increased levels of reactive oxygen species (ROS) and the release of multiple proinflammatory cytokines, such as tumor necrosis factor-alpha (TNF-α), interleukin-1 (IL-1), and interleukin-6 (IL-6) [43,44]. Notably, oxidative stress plays a crucial role in COVID-19, with excessive ROS generation contributing to the onset of cytokine storms. Cytokine storm is a major factor in the progression of severe COVID-19 infection, characterized by uncontrolled production and release of various cytokines, including those influenced by PM2.5 exposure [45]. This dysregulated immune response can trigger a cascade of cellular death, eventually leading to multi-organ failure and life-threatening complications. Considering these findings, we propose that PM2.5 exposure may exacerbate immune response and oxidative stress in COVID-19 patients, potentially increasing the risk of severe infection and mortality. Understanding the impact of PM2.5 on the excessive immune response during COVID-19 can help inform public health strategies and guide interventions to mitigate the adverse health effects of air pollution on COVID-19. It is noteworthy that particulate matter not only affects human physiology directly, but also indirectly influences atmospheric conditions, which can in turn affect how the human body responds to viral infections [46,47]. Further research on the atmospheric environment-human-virus interplay could enhance our understanding of the health impacts associated with particulate matter.

We conducted a two-sample MR analysis to investigate the causal relationship between air pollution exposure and COVID-19 outcomes. While RCTs are generally considered to provide more robust and conclusive evidence, conducting large-scale clinical trials that investigate the effects of different pollution levels can pose significant challenges in terms of both budgetary and ethical considerations. MR analysis offers advantages over traditional observational studies, as it is less susceptible to confounding and measurement error. When the three key assumptions are met (as outlined in Methods), the IVW model can provide the most accurate estimate of the causal effect [48]. Alternative methods, such as the weighted median and MR-Egger approaches, are more robust to horizontal pleiotropy but generally have lower statistical power compared to the IVW method [21,49]. An insufficient number of SNPs was identified for PM2.5–10 and PM10 exposure using the standard significance level (5 × 10^−8^). Thus, we relaxed the *P*-value threshold for selection of instrument variables in order to expand the number of SNPs used in these two exposures. By evaluating various sensitivity analysis methods, such as the RAPS and GSMR approaches, we aimed to mitigate potential shortcomings associated with relaxed *P*-value thresholds and the resulting pleiotropy and weak instrument bias [25]. Since we used a relatively relaxed significance level for IV screening, the RAPS method can better address the bias caused by potential weak instruments and pleiotropy (both systematic and idiosyncratic) [50]. The GSMR analysis is an innovative approach that utilizes a generalized least-square method to assess causal effects by factoring in potential LD connectivity and undetected pleiotropy of the SNPs, as SNPs with potential pleiotropic effects would be detected by HEIDI-outlier test [23]. A previous study has demonstrated that an integrated MR approach, which combined various methods including RAPS and GSMR, can optimize sensitivity analysis and improve the validity of causal estimates [51]. The PhenoScanner search results revealed that some IVs were associated with haematological traits. However, according to the MR results from a previous study, among 34 haematological traits [52], only the monocyte percentage of white cells showed a significant causal association with COVID-19 severity, while no haematological trait was found to be related to the risk of COVID-19 hospitalization [53]. Importantly, none of the IVs used in our study was linked to this specific haematological trait; therefore, no SNPs were excluded due to potential associations with confounding factors.

There are a few limitations to our study. First, it is recommended to ensure that the exposure and outcome studies used in two-sample MR analysis do not involve overlapping participants. In this particular study, we could not ascertain the extent of overlap; however, any potential bias stemming from sample overlap can be mitigated by utilizing robust IVs, such as those with an F-statistic higher than 10. The MR analysis can provide more accurate and reliable causal inferences by employing robust instruments, reducing the likelihood of spurious associations due to sample overlap [54]. Second, while our study had moderate statistical power to detect effects on COVID-19 severity and hospitalization, the power to detect susceptibility was relatively low. This underscores the importance of conducting more comprehensive studies with larger sample sizes. Nevertheless, our study maintained over 80% power to identify a theoretical OR of 1.5 for COVID-19 susceptibility. Furthermore, it is plausible that exposure to pollution at different stages of SARS-CoV-2 infection duration may have varying impact to the COVID-19 outcomes. However, it is important to note that MR analysis reflects lifelong effects and cannot discern the specific duration of exposure and its influence on the outcomes, necessitating further investigation through methods such as RCTs. Finally, the compatibility of exposure and outcome data sources was challenged, as the ESCAPE estimated air pollution levels was only applicable with good confidence within the Greater London area, while the participants in the COVID-19 Host Genetics Initiative were spread all over Europe, with the UK being the predominant data source [15]. Therefore, the generalizability of our findings requires further validation.

## 5 Conclusion

In summary, our study is the first to assess causal relationship between air pollution and COVID-19 using multiple MR methods. MR results suggested that PM2.5 concentration and absorbance, rather than larger particulate matter (PM10 and PM2.5-10), can significantly increase the risk of COVID-19 severity and hospitalization. This highlights the crucial role of addressing PM2.5 air pollution to mitigate the adverse health effects on COVID-19 patients. Efforts to reduce PM2.5 levels can not only improve overall air quality but might also contribute to a reduction in the severity of respiratory diseases.

## Funding

This work was supported by the Key Project of Hunan Provincial Science and Technology Innovation [no. 2020SK1013]; the 10.13039/501100001809National Natural Science Foundation of China [no. 82070356, 82270337]; the 10.13039/501100004735Hunan Provincial Natural Science Foundation of China [no. 2021JJ30033, 2021JJ40870]; the Hunan Provincial Health Commission Scientific Research Project [no. 20201302]; and the Fundamental Research Funds for the Central Universities of 10.13039/501100002822Central South University [no. 2022ZZTS0863].

## Data availability statement

Publicly available datasets were used in MR analysis. The GWAS datasets can be found here: https://gwas.mrcieu.ac.uk/datasets/and https://covid19hg.org/. The exposure dataset IDs were ukb-b-10817 for PM2.5 concentration, ukb-b-11312 for PM2.5 absorbance, ukb-b-12963 for PM2.5-10 concentration, and ukb-b-18469 for PM10 concentration.

## CRediT authorship contribution statement

**Jiayi Zhu:** Writing – original draft, Visualization, Methodology, Investigation, Funding acquisition, Formal analysis, Conceptualization. **Yong Zhou:** Writing – review & editing, Resources, Data curation. **Qiuzhen Lin:** Writing – review & editing, Resources, Data curation. **Keke Wu:** Writing – review & editing, Resources, Data curation. **Yingxu Ma:** Writing – review & editing, Resources, Data curation. **Chan Liu:** Writing – review & editing, Resources, Data curation. **Na Liu:** Writing – review & editing, Resources, Funding acquisition, Data curation. **Tao Tu:** Writing – review & editing, Supervision, Project administration, Funding acquisition, Data curation, Conceptualization. **Qiming Liu:** Writing – review & editing, Supervision, Project administration, Funding acquisition, Data curation, Conceptualization.

## Declaration of competing interest

The authors declare that they have no known competing financial interests or personal relationships that could have appeared to influence the work reported in this paper.
