# Supplementary material for: Causal relationship between particulate matter and COVID-19 risk: A mendelian randomization study
Source: Heliyon. 2024 Feb 24;10(5):e27083. doi: 10.1016/j.heliyon.2024.e27083 (PMC10909784; doi:10.1016/j.heliyon.2024.e27083)
Supplement: Multimedia component 1 [file mmc1.docx]

**Supplementary Table 1 Power analysis (two-sided α=0.05) for Mendelian randomization analysis.** OR were estimated by IVW method. A power analysis was also conducted for each MR analysis using theoretical OR value of 1.5. OR, odd ratio.

| Exposure | Outcome | Sample Size | Number of Cases | OR | R2 of instruments (%) | Power (Observed Effect) | Power (Theoretical Effect 1.5) |
| --- | --- | --- | --- | --- | --- | --- | --- |
| PM2.5 concentration | Severity | 1086211 | 13769 | 3.29 | 0.069 | 95.50% | 23.60% |
|  | Hospitalization | 2095324 | 32519 | 1.91 | 0.069 | 86.10% | 47.90% |
|  | Susceptibility | 2597856 | 122616 | 1.02 | 0.069 | 3.90% | 95.00% |
| PM2.5 absorbance | Severity | 1086211 | 13769 | 5.62 | 0.041 | 98.20% | 15.70% |
|  | Hospitalization | 2095324 | 32519 | 2.83 | 0.041 | 96.40% | 31.00% |
|  | Susceptibility | 2597856 | 122616 | 1.15 | 0.041 | 15.70% | 79.90% |
| PM2.5-10 concentration | Severity | 1086211 | 13769 | 1.78 | 0.042 | 28.10% | 16.20% |
|  | Hospitalization | 2095324 | 32519 | 2.09 | 0.042 | 77.50% | 32.00% |
|  | Susceptibility | 2597856 | 122616 | 1.21 | 0.042 | 26.20% | 81.40% |
| PM10 concentration | Severity | 1086211 | 13769 | 3.74 | 0.049 | 92.70% | 18.10% |
|  | Hospitalization | 2095324 | 32519 | 2.24 | 0.049 | 89.40% | 36.40% |
|  | Susceptibility | 2597856 | 122616 | 1.03 | 0.049 | 4.20% | 87.00% |

**Supplementary Table 2 Characteristics of instrumental variables.**

| Exposure | Rsid | Chromosome | Position | Effect allele | Other allele | F-statistics |
| --- | --- | --- | --- | --- | --- | --- |
| PM2.5 concentration | rs6749467 | 2 | 343517 | A | G | 32.23 |
|  | rs1372504 | 5 | 103749428 | A | G | 30.67 |
|  | rs12203592 | 6 | 396321 | T | C | 69.92 |
|  | rs77255816 | 6 | 20833602 | T | C | 30.04 |
|  | rs114708313 | 6 | 31329004 | T | A | 30.08 |
|  | rs77205736 | 8 | 10153460 | T | C | 31.40 |
|  | rs1537371 | 9 | 22099568 | A | C | 33.15 |
|  | rs72642437 | 18 | 45920421 | T | C | 35.12 |
| PM2.5 absorbance | rs4915350 | 1 | 199204067 | C | A | 33.93 |
|  | rs12203592 | 6 | 396321 | T | C | 41.54 |
|  | rs59727727 | 6 | 31329797 | C | T | 30.82 |
|  | rs79475047 | 6 | 20992197 | C | T | 36.43 |
|  | rs77205736 | 8 | 10153460 | T | C | 29.91 |
| PM2.5-10 concentration | rs78060907 | 5 | 37945741 | A | C | 24.44 |
|  | rs9497937 | 6 | 148423453 | A | C | 24.56 |
|  | rs1706918 | 7 | 105272307 | A | G | 24.91 |
|  | rs118101191 | 7 | 111672596 | T | G | 29.21 |
|  | rs1157546 | 8 | 18777006 | C | T | 25.20 |
|  | rs11621531 | 14 | 103577789 | A | G | 26.56 |
|  | rs12462492 | 19 | 22710669 | T | G | 24.64 |
| PM10 concentration | rs117671171 | 6 | 159246615 | T | C | 25.50 |
|  | rs12203592 | 6 | 396321 | T | C | 25.68 |
|  | rs12192953 | 6 | 94818099 | T | C | 27.49 |
|  | rs57048268 | 7 | 151623218 | C | A | 28.07 |
|  | rs149130673 | 11 | 83722884 | C | T | 24.42 |
|  | rs79037220 | 13 | 50507971 | A | G | 25.04 |
|  | rs80230137 | 14 | 48380718 | G | A | 26.03 |
|  | rs4815138 | 20 | 286487 | A | G | 26.89 |

**Supplementary Table 3 Complete mendelian randomization estimates between PM2.5 concentration and risk of COVID-19.** IVW, inverse variance weighted; FE-IVW, fixed effects inverse variance weighted; MRE-IVW, multiplicative random effects inverse variance weighted.

| Method | Severity | | |  | Hospitalization | | |  | Susceptibility | | |
| --- | --- | --- | --- | --- | --- | --- | --- | --- | --- | --- | --- |
|  | Beta | SE | P |  | Beta | SE | P |  | Beta | SE | P |
| MR Egger | 1.22 | 0.91 | 0.2274 |  | 0.78 | 0.69 | 0.3005 |  | 0.11 | 0.22 | 0.6205 |
| Weighted median | 1.01 | 0.53 | 0.0574 |  | 0.57 | 0.37 | 0.1251 |  | -0.02 | 0.15 | 0.8944 |
| IVW | 1.19 | 0.41 | 0.0036 |  | 0.65 | 0.30 | 0.0318 |  | 0.02 | 0.12 | 0.8472 |
| FE-IVW | 1.19 | 0.41 | 0.0036 |  | 0.65 | 0.27 | 0.0152 |  | 0.02 | 0.12 | 0.8472 |
| MRE-IVW | 1.19 | 0.40 | 0.0029 |  | 0.65 | 0.30 | 0.0318 |  | 0.02 | 0.11 | 0.8429 |
| Simple mode | 1.13 | 0.89 | 0.2440 |  | 0.68 | 0.54 | 0.2493 |  | -0.10 | 0.22 | 0.6702 |
| Weighted mode | 1.10 | 0.83 | 0.2296 |  | 0.59 | 0.51 | 0.2858 |  | -0.01 | 0.21 | 0.9804 |
| Maximum likelihood | 1.24 | 0.42 | 0.0033 |  | 0.67 | 0.28 | 0.0155 |  | 0.02 | 0.12 | 0.8449 |
| RAPS | 1.22 | 0.43 | 0.0048 |  | 0.67 | 0.28 | 0.0173 |  | 0.02 | 0.12 | 0.8502 |

**Supplementary Table 4 Complete mendelian randomization estimates between PM2.5 absorbance and risk of COVID-19.**

| Method | Severity | | |  | Hospitalization | | |  | Susceptibility | | |
| --- | --- | --- | --- | --- | --- | --- | --- | --- | --- | --- | --- |
|  | Beta | SE | P |  | Beta | SE | P |  | Beta | SE | P |
| MR Egger | 0.19 | 1.46 | 0.9072 |  | -0.59 | 1.64 | 0.7434 |  | -0.44 | 0.56 | 0.4904 |
| Weighted median | 1.10 | 0.73 | 0.1308 |  | 0.08 | 0.50 | 0.8678 |  | -0.02 | 0.25 | 0.9367 |
| IVW | 1.73 | 0.66 | 0.0089 |  | 1.04 | 0.70 | 0.1387 |  | 0.14 | 0.24 | 0.5683 |
| FE-IVW | 1.73 | 0.53 | 0.0012 |  | 1.04 | 0.35 | 0.0033 |  | 0.14 | 0.17 | 0.4160 |
| MRE-IVW | 1.73 | 0.66 | 0.0089 |  | 1.04 | 0.70 | 0.1387 |  | 0.14 | 0.24 | 0.5683 |
| Simple mode | 0.89 | 1.06 | 0.4490 |  | -0.06 | 0.68 | 0.9374 |  | 0.05 | 0.36 | 0.9053 |
| Weighted mode | 0.82 | 0.90 | 0.4123 |  | -0.08 | 0.59 | 0.8996 |  | 0.05 | 0.34 | 0.8985 |
| Maximum likelihood | 1.80 | 0.56 | 0.0014 |  | 1.15 | 0.39 | 0.0029 |  | 0.14 | 0.17 | 0.4077 |
| RAPS | 1.80 | 0.57 | 0.0015 |  | 1.15 | 0.37 | 0.0018 |  | 0.14 | 0.17 | 0.4094 |

**Supplementary Table 5 Complete mendelian randomization estimates between PM2.5-10 concentration and risk of COVID-19.**

| Method | Severity | | |  | Hospitalization | | |  | Susceptibility | | |
| --- | --- | --- | --- | --- | --- | --- | --- | --- | --- | --- | --- |
|  | Beta | SE | P |  | Beta | SE | P |  | Beta | SE | P |
| MR Egger | -0.53 | 1.28 | 0.6937 |  | -0.06 | 0.92 | 0.9527 |  | -0.51 | 0.51 | 0.3618 |
| Weighted median | 0.68 | 0.70 | 0.3331 |  | 0.48 | 0.49 | 0.3303 |  | 0.11 | 0.24 | 0.6392 |
| IVW | 0.58 | 0.53 | 0.2815 |  | 0.74 | 0.37 | 0.0469 |  | 0.19 | 0.23 | 0.4045 |
| FE-IVW | 0.58 | 0.53 | 0.2815 |  | 0.74 | 0.36 | 0.0425 |  | 0.19 | 0.17 | 0.2632 |
| MRE-IVW | 0.58 | 0.43 | 0.1806 |  | 0.74 | 0.37 | 0.0469 |  | 0.19 | 0.23 | 0.4045 |
| Simple mode | 0.78 | 1.08 | 0.4985 |  | 0.36 | 0.77 | 0.6596 |  | 0.08 | 0.43 | 0.8645 |
| Weighted mode | 0.76 | 1.15 | 0.5335 |  | 0.28 | 0.73 | 0.7105 |  | 0.08 | 0.43 | 0.8623 |
| Maximum likelihood | 0.59 | 0.54 | 0.2775 |  | 0.76 | 0.37 | 0.0408 |  | 0.20 | 0.17 | 0.2524 |
| RAPS | 0.59 | 0.56 | 0.2945 |  | 0.76 | 0.38 | 0.0457 |  | 0.20 | 0.17 | 0.2489 |

**Supplementary Table 6 Complete mendelian randomization estimates between PM10 concentration and risk of COVID-19.**

| Method | Severity | | |  | Hospitalization | | |  | Susceptibility | | |
| --- | --- | --- | --- | --- | --- | --- | --- | --- | --- | --- | --- |
|  | Beta | SE | P |  | Beta | SE | P |  | Beta | SE | P |
| MR Egger | 0.76 | 1.02 | 0.4841 |  | -0.01 | 0.79 | 0.9857 |  | -0.26 | 0.34 | 0.4793 |
| Weighted median | 1.35 | 0.65 | 0.0378 |  | 0.35 | 0.44 | 0.4265 |  | 0.02 | 0.21 | 0.9093 |
| IVW | 1.32 | 0.53 | 0.0136 |  | 0.81 | 0.44 | 0.0656 |  | 0.03 | 0.18 | 0.8668 |
| FE-IVW | 1.32 | 0.46 | 0.0041 |  | 0.81 | 0.32 | 0.0108 |  | 0.03 | 0.15 | 0.8438 |
| MRE-IVW | 1.32 | 0.53 | 0.0136 |  | 0.81 | 0.44 | 0.0656 |  | 0.03 | 0.18 | 0.8668 |
| Simple mode | 1.37 | 0.84 | 0.1481 |  | 0.18 | 0.62 | 0.7836 |  | 0.12 | 0.33 | 0.7392 |
| Weighted mode | 1.32 | 0.80 | 0.1419 |  | 0.20 | 0.63 | 0.7545 |  | 0.11 | 0.31 | 0.7377 |
| Maximum likelihood | 1.38 | 0.48 | 0.0042 |  | 0.84 | 0.33 | 0.0115 |  | 0.03 | 0.16 | 0.8404 |
| RAPS | 1.38 | 0.49 | 0.0048 |  | 0.85 | 0.33 | 0.0105 |  | 0.03 | 0.16 | 0.8426 |
