# Supplementary material for: Causal relationship between particulate matter and COVID-19 risk: A mendelian randomization study
Source: Heliyon. 2024 Feb 24;10(5):e27083. doi: 10.1016/j.heliyon.2024.e27083 (PMC10909784; doi:10.1016/j.heliyon.2024.e27083)
Supplement: Multimedia component 2 [file mmc2.docx]

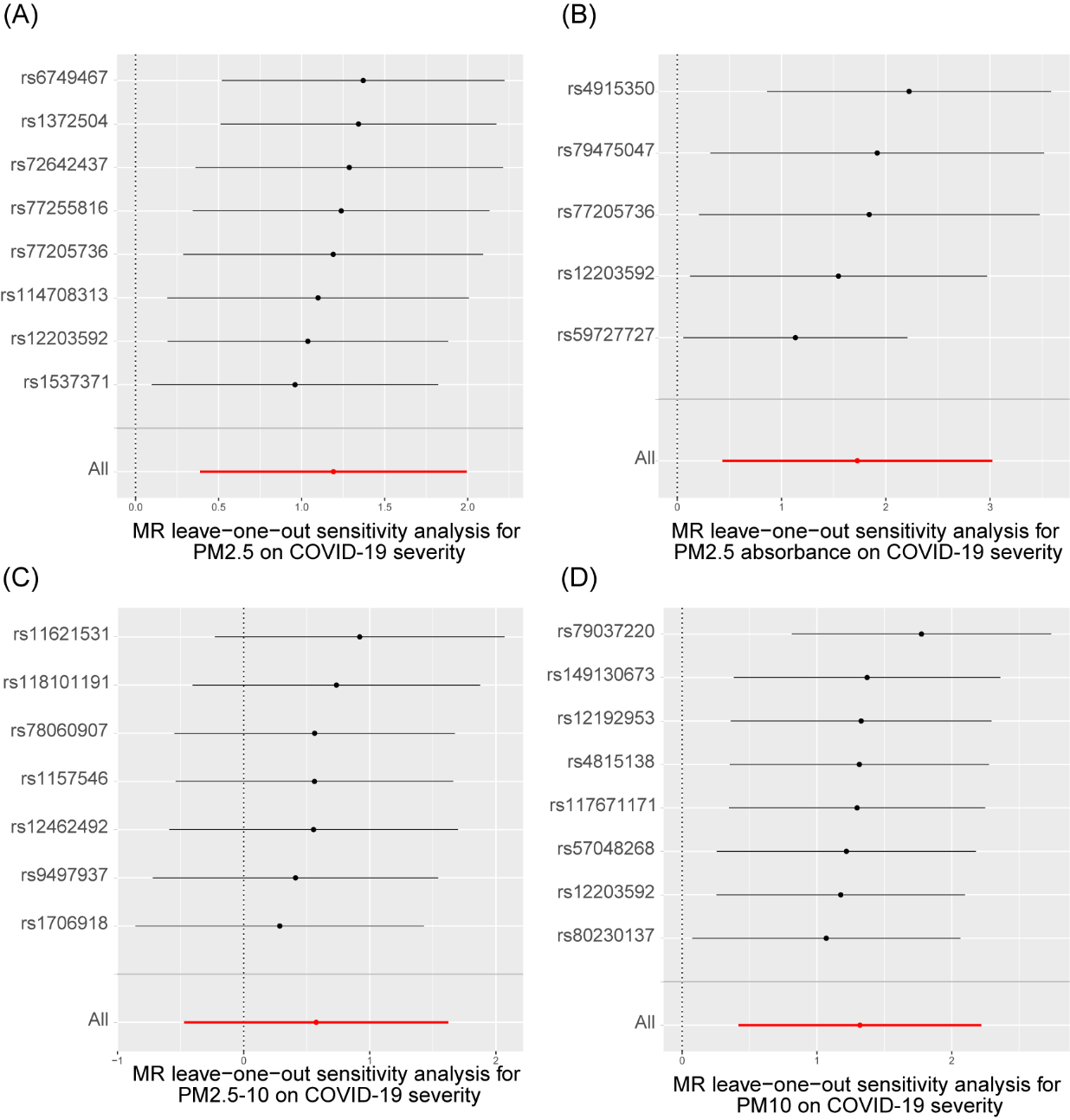


**Supplementary Figure 1 The leave-one-out analysis for each particulate matter exposure and COVID-19 severity.** No instrumental variables were found to influence overall MR analysis substantially.
